# High casein kinase 1 epsilon levels are correlated with better prognosis in subsets of patients with breast cancer

**DOI:** 10.18632/oncotarget.4850

**Published:** 2015-07-13

**Authors:** Jose Luis Lopez-Guerra, Eva M. Verdugo-Sivianes, Daniel Otero-Albiol, Begoña Vieites, Maria J. Ortiz-Gordillo, Jose M. De León, Juan M. Praena-Fernandez, Juan J. Marin, Amancio Carnero

**Affiliations:** ^1^ Instituto de Biomedicina de Sevilla (IBIS/HUVR/CSIC/Universidad de Sevilla), Seville, Spain; ^2^ Department of Radiation Oncology, University Hospital Virgen del Rocío, Seville, Spain; ^3^ Department of Pathology, University Hospital Virgen Del Rocío, Seville, Spain; ^4^ Unidad Funcional de Patología Mamaria, University Hospital Virgen Del Rocío, Seville, Spain; ^5^ Methodology Unit- Fundación Pública Andaluza para la Gestión de la Investigación en Salud de Sevilla, Virgen del Rocío University Hospital, Seville, Spain; ^6^ Department of Preventive Medicine and Public Health, Seville University, Seville, Spain; ^7^ Consejo Superior de Investigaciones Científicas

**Keywords:** breast cancer, casein kinase 1 epsilon (CK1ε), relapse, disease-free survival, outcome

## Abstract

Reliable biological markers that predict breast cancer (BC) outcomes after multidisciplinary therapy have not been fully elucidated. We investigated the association between casein kinase 1 epsilon (CK1ε) and the risk of recurrence in patients with BC. Using 168 available tumor samples from patients with BC treated with surgery +/− chemo(radio)therapy, we scored the CK1ε expression as high (≥1.5) or low (<1.5) using an immunohistochemical method. Kaplan-Meier analysis was performed to assess the risk of relapse, and Cox proportional hazards analyses were utilized to evaluate the effect of CK1ε expression on this risk. The median age at diagnosis was 60 years (range 35-96). A total of 58% of the patients underwent breast conservation surgery, while 42% underwent mastectomy. Adjuvant chemotherapy and radiation therapy were administered in 101 (60%) and 137 cases (82%), respectively. Relapse was observed in 24 patients (14%). Multivariate analysis found high expression of CK1ε to be associated with a statistically significant higher disease-free survival (DFS) in BC patients with wild-type p53 (Hazard ratio [HR] = 0.33; 95% CI, 0.12-0.91; *P* = 0.018) or poor histological differentiation ([HR] = 0.34; 95% CI, 0.12-0.94; *P* = 0.039) or in those without adjuvant chemotherapy ([HR] = 0.11; 95% CI, 0.01-0.97; *P* = 0.006). Our data indicate that CK1ε expression is associated with DFS in BC patients with wild-type p53 or poor histological differentiation or in those without adjuvant chemotherapy and thus may serve as a predictor of recurrence in these subsets of patients.

## INTRODUCTION

Breast cancer is the second leading cause of cancer death in women, responsible for approximately 3% of deaths. Death rates from breast cancer have been declining since approximately 1989(American Cancer society, http://www.cancer.org) believed to be the result of earlier detection through screening and increased awareness, as well as improved treatment. Depending on the stage of the tumor, local or systemic therapies are administered. Patients who have no detectable cancer after surgery are often given adjuvant therapy to help prevent the cancer from relapsing. In the setting of multidisciplinary therapies for breast cancer (BC), relapses are a severe event, leading to hospitalizations, second surgeries, or the initiation of systemic treatments affecting the survival outcome. Both systemic therapy, such as chemotherapy, hormone therapy and targeted therapy, and radiation can be used as adjuvant therapy. Most, but not all, patients benefit from adjuvant therapy. How much the patient might benefit depends on the stage and characteristics of the cancer and the type of surgery. Recent studies [1, 2] have shown that specific receptors predict relapse in BC patients and have been used as targets to improve the outcome. For example, a review of 119 BC patients receiving trastuzumab for human epidermal growth factor receptor-2 (HER2)-positive metastatic disease, indicated that those receiving taxanes with trastuzumab had a median overall survival that was significantly longer compared to other first-line partners [2].

However, beyond these tumor-staging endpoints, there is no reliable information that can predict which tumors treated with adjuvant chemotherapy are more likely to relapse or to obtain a benefit. Although some clinical and pathological parameters have been included in tumor response models to guide therapy and prevent relapse, the predictive value of these parameters is limited, as demonstrated by the observation that patients with similar characteristics often have contrasting tumor responses. One way to improve on these models would be to include biological markers representing tumor tissue sensitivity

Casein kinase 1 (CK1) is a member of a family of serine/threonine protein kinases. CK1 kinases exist in at least seven isoforms (α, β, γ1–3, δ and ε) in mammals [3–5]. CK1 kinases phosphorylate various substrates that play vital roles in diverse physiological processes such as DNA repair, cell cycle progression, cytokinesis, differentiation and apoptosis [4–6]. CK1-epsilon (CK1ε) is a protein product of the *CSNK1E* gene. CK1ε has been shown to be essential in regulating cell division and tumor growth in human pancreatic and colon adenocarcinoma cells and in salivary gland cancer by phosphorylating key proteins in the Wnt signaling pathway [7–10].

Changes in CK1ε expression and activity, as well as the occurrence of mutations within the coding region of CK1ε, have been reported in various cancers, including breast and ovarian cancers [4–6, 11, 12]. Our study investigates whether differences in CK1ε expression are associated with clinicopathological and molecular parameters in patients with BC who receive surgery +/− chemo(radio)therapy. This information could be determined before therapy and incorporated into tumor tissue response models to plan the treatment for individual patients.

## RESULTS

### Overexpression of active CK1ε enhances growth of tumor cells *in vitro* and sensitivity to UV exposure

**Figure 1:**
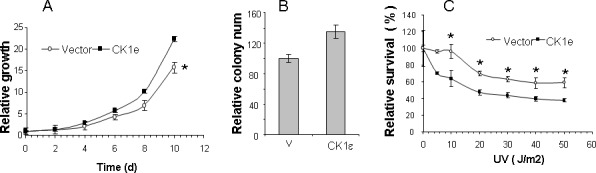
Overexpression of active CK1ε enhances growth of tumor cells in vitro and sensitivity to UV exposure **A.** Active CK1ε increased the growth of mammary tumor cells. Growth curve of parental T47D cells expressing an empty vector (vector) or myristoylated CK1ε expressing mass culture (CK1ε) in T47D. Values are expressed as the percentage of cell growth. The data are presented as the mean of triplicate samples; bars, ± SD. **B.** Colony assay of T47D cells expressing an empty vector (vector) or myristoylated CK1ε expressing mass culture (CK1ε) in T47D. Values are represented as the percentage of number of colonies. Parental T47D control cells represent 100% of cell growth. The data are presented as the mean of triplicate samples; bars, ± SD. **C.** Response to UV irradiation in T47D cells expressing an empty vector (vector) or myristoylated CK1ε expressing mass culture (CK1ε) in T47D. Dose response effect on cells 48 hrs after UV irradiation. Values are expressed as the percentage of live cells. Untreated control cells represent 100% of the cells. The Student T test was used to compare the statistical significance of the differences among the survival curves of parental cells versus the clones. * = *p* < 0.05.

We recovered the full-length *CSNK1E* gene from one gain-of-function genetic screening event to identify genes that are able to alter the cellular response to physiological signals and provide a selective advantage once tumorigenesis has begun [13]. The CK1ε promotes oncogenic transformation in multiple cell types, including immortal non-tumoral human mammary epithelial cells HEMCs [14]; however, transformation only occurs if myristoylated (active) CK1ε is expressed because wild-type CK1ε does not seem to contribute to tumorigenesis [14]. Therefore, we tested whether myristoylated CK1ε induced an enhancement of tumorigenic properties in mammary tumor cells. We expressed myristoylated CK1ε (see M&M) or empty vectors in ductal adenocarcinoma T47D cells. We found that myristoylated CK1ε induced a significant increase in growth and colony forming efficiency in these cells (Figure 1A and 1B). Furthermore, we subjected these cells to different doses of UV irradiation as a surrogate for radiotherapy. In this setting, the expression of myristoylated CK1ε induced some sensitivity to irradiation (Figure 1C).

### CK1ε levels in breast tumors

To determine the relevance of CK1ε in human mammary tumors we analyzed tumor samples from 168 BC patients (Table 1). The median age was 60 years (a range of 35–96 years). There were 63 patients with stage I, 68 with stage II and 37 with stage III/IV. Twenty-three tumors were well differentiated, 59 were moderately differentiated, and 84 were poorly differentiated. The median follow-up was 70 months with a median disease-free survival (DFS) of 65 months (range, 1-76 months). The five-year overall survival (OS) rate was 88%. Fifty-eight percent of the patients underwent conservative breast surgery, while forty-two percent were treated by mastectomy. Adjuvant therapy was administered according to individual considerations. Chemotherapy and/or radiation therapy were delivered in 60% and 82% of patients, respectively. Relapse was observed in 24 patients (14%).

**Table 1:** Patient characteristics

Characteristics	No. of Patients (%)
	(*N* = 168)
Age (years)	
Median (range)	60 (35 to 96)
Type	.
Invasive ductal carcinoma	151 (90)
Invasive lobular carcinoma	8 (5)
Mucinous carcinoma	4 (2)
Metaplastic carcinoma	1 (0.5)
Micropapillary carcinoma	3 (2)
Medullary carcinoma	1 (0.5)
Grade (N=168)	
Well differentiated	23 (14)
Moderately differentiated	59 (35)
Poorly differentiated	84 (50)
Undifferentiated	2 (1)
Estrogen receptors	
Yes	133 (79)
No	35 (21)
Progesterone receptors	
Yes	119 (71)
No	49 (29)
Human epidermal growth factor receptor	
Yes	11 (6)
No	157 (94)
Ki-67	
Median (range)	18 (5-70)
Stage	
I	63 (37)
II	68 (41)
III/IV	37 (22)
Chemotherapy	
Yes	101 (60)
No	67 (40)
Type of surgery	
Conservative	97 (58)
Mastectomy	71 (42)
Hormone therapy	
Yes	125 (75)
No	43 (25)
Radiation therapy	
Yes	137 (82)
No	31 (18)
CK1ε	
High expression (≥1.5)	96 (57)
Low expression (<1.5)	72 (43)
Luminal A	
Yes	120 (71)
No	48 (29)
Luminal B	
Yes	13 (8)
No	155 (92)
Luminal HER2	
Yes	11 (7)
No	157 (93)
Triple-negative	
Yes	24 (14)
No	144 (86)
Recurrence	
Distant	22 (13)
Loco-regional	4 (2)
Status	
Alive	145 (86)
Death	23 (14)
Follow-up (months)	
Median (range)	70 (10-77)

**Figure 2:**
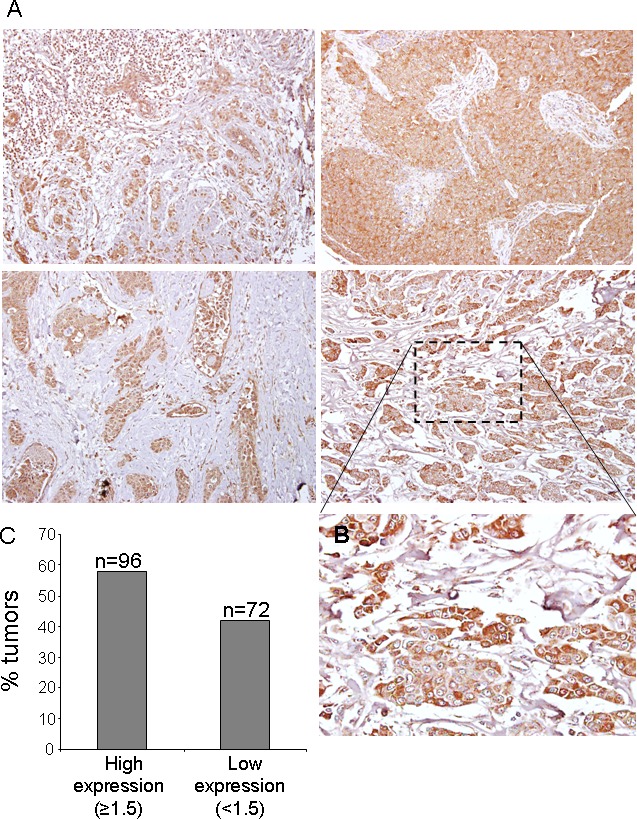
CK1ε expression in human mammary tumors **A.** Representative images of CK1ε in human mammary tumors. **B.** Inset amplification of tumor staining. **C.** Graph showing the percentage of tumors with high (score >1.5) or low (score <1.5) levels of CK1ε.

**Figure 3:**
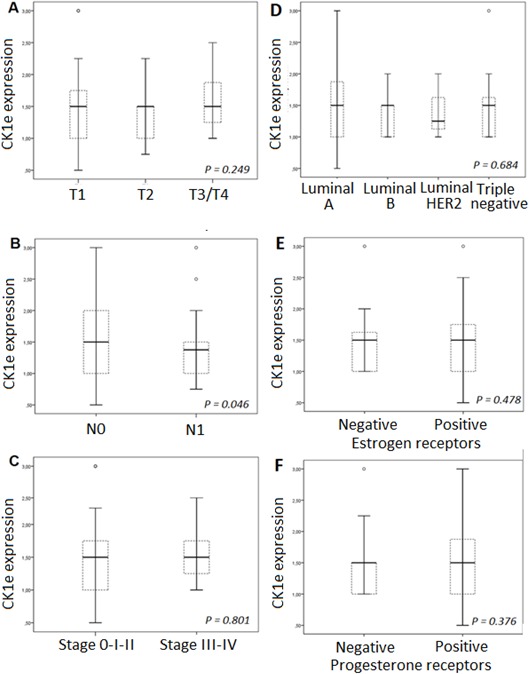
CK1ε expression according to **A.** molecular subtypes, **B.** estrogen receptors, and **C.** progesterone receptors; **D.** T classification, **E.** N status, and **F.** tumor stage.

CK1ε expression, mainly cytoplasmic (see Figure 2), was considered low (<1.5) in 72 patients and high (≥1.5) in 96 patients (Figure 2), indicating that 57% of the patients showed high levels of CK1ε. The staining was homogeneous for most samples, with some samples with some minimal heterogeneity in signal intensity among tumor cells. Those with positive nodal status had a narrower CK1ε expression range than those without nodal involvement (*P* = 0.025, Figure 3B). However, there was no difference in CK1ε expression according to stage (*P* = 0.099, Figure 3C) or molecular subtype (*P* = 0.648, Figure 3D). Similar findings were observed when CK1ε expression was evaluated with regard to hormonal receptors (*P* = 0.478 and 0.373, Figures 3E and 3F).

### High CK1ε levels correlated with better prognosis in subset of patients with breast tumors

**Figure 4:**
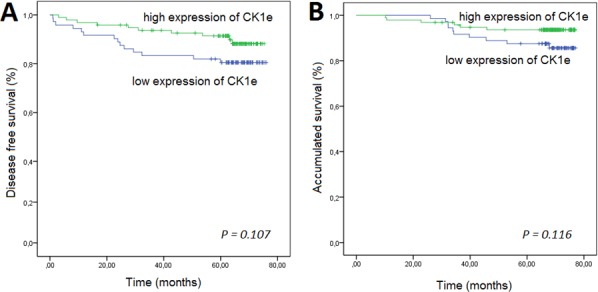
Kaplan-Meier survival curves for breast cancer patients who were classified with either low or high CK1ε expression High CK1ε expression was associated (log-rank) with better **A.** disease-free survival and **B.** overall survival.

The five-year DFS rate was 81% for the group with low CK1ε expression and 92% for the group with higher expression (*P* = 0.107, Figure 4A). Although not significant, there is a trend towards the relevance of levels of CK1ε in DFS in mammary tumor patients. A similar trend was observed for the total survival rate (*P* = 0.116, Figure 4B).

**Figure 5:**
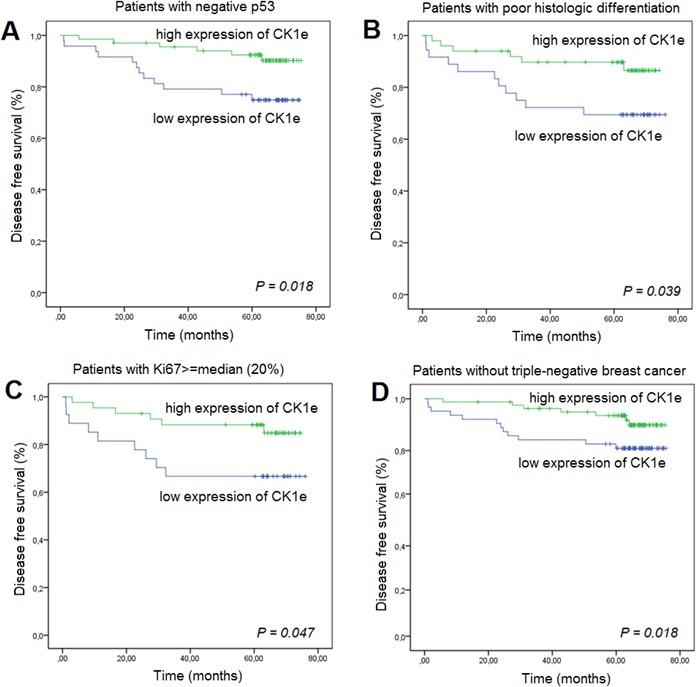
Kaplan-Meier survival curves for breast cancer patients who were classified with either low or high CK1ε expression High CK1ε expression was associated (log-rank) with better prognosis as measured by disease-free survival in patients with **A.** negative p53, **B.** poor histologic differentiation, **C.** Ki67 ≥median, or **D.** triple-negative breast cancer.

The correlation between the expression levels of CK1ε and clinical parameters is summarized in Table 2. Univariate Cox proportional hazard analyses of the data for DFS showed that higher CK1ε expression is associated with an increase in DFS in BC patients with negative (wild type) p53 (HR = 0.32, 95% CI, 0.12 to 0.86, *P* = 0.018; Table 2), poor histologic differentiation (HR = 0.36, 95% CI, 0.13 to 0.98, *P* = 0.039), Ki67 ≥median (HR = 0.36, 95% CI, 0.13 to 1.03, *P* = 0.047), or a triple-negative subtype (HR = 0.42, 95% CI, 0.16 to 1.08, *P* = 0.018) (Figure 5). This effect was virtually unchanged after adjusting for other confusing factors (i.e., pathologic stage) by multivariate analysis in BC patients with negative p53 (HR = 0.33, 95% CI, 0.12 to 0.91, *P* = 0.032) or poor histologic differentiation (HR = 0.32, 95% CI, 0.12 to 0.94, *P* = 0.032).

**Table 2:** Univariate and multivariate analyses of associations between clinicopathologic factors and disease-free survival (DFS) in patients with significant/marginal association found between CK1ε and DFS in the univariate analysis

Subset	Characteristic	Univariate Analysis†				Multivariate Analysis††		
		No.*	HR	CI	P	HR	CI	P
Patients with negative p53	Histologic differentiation							
	Well/Moderate	63/6						
	Poor	53/12	2.67	1.01-7.12	0.049	2.65	0.97-7.19	0.056
	Pathologic Stage							
	I/II	96/10						
	III/IV	20/8	4.44	1.74-11.28	0.002	2.94	1.11-7.80	0.030
	Surgical procedure							
	Lumpectomy	67/6						
	Mastectomy	49/12	2.95	1.11-7.87	0.031	2.03	0.73-5.59	0.170
	CK1ε							
	<1,5	48/12						
	≥1,5	68/6	0,32	0,12-0,86	0,025	0.32	0.12-0.91	0.032
Patients with age ≥median	Pathologic Stage							
	I/II	68/8						
	III/IV	16/6	3.07	1.09-8.65	0.033	1.45	0.49-4.30	0.499
	Surgical procedure							
	Lumpectomy	47/3						
	Mastectomy	37/12	5.83	1.64-20.72	0.006	5.22	1.39-19.54	0.014
	CK1ε							
	<1,5	40/11						
	≥1,5	44/4	0.32	0.10-1.01	0.050	0.33	0.10-1.05	0.060
Patients with poor histologic differentiation	Pathologic Stage							
	I/II	62/7						
	III/IV	24/10	4.09	1.55-10.75	0.004	2.78	1.00-7.74	0.50
	Surgical procedure							
	Lumpectomy	42/4						
	Mastectomy	44/13	3.39	1.10-10.42	0.033	2.75	0.83-9.06	0.095
	CK1ε							
	<1,5	36/11						
	≥1,5	50/6	0.36	0.13-0.98	0.048	0.34	0.12-0.94	0.038
Patients with Ki67 ≥median	Pathologic Stage							
	I/II	50/7						
	III/IV	20/8	3.09	1.12-8.54	0.029	2.88	1.04-7.98	0.041
	CK1ε							
	<1,5	27/9						
	≥1,5	43/6	0.36	0.13-1.03	0.057	0.39	0.14-1.11	0.080
Patients without chemotherapy	Pathologic Stage							
	I/II	57/4						
	III/IV	10/5	8.01	2.13-30.03	0.002	4.05	1.01-16.19	0.0437
	Ki67 (%)							
	<median	45/3						
	≥median	20/6	5.21	1.30-20.85	0.020	4.45	1.06-18.61	0.041
	CK1ε							
	<1,5	30/8						
	≥1,5	37/1	0.09	0.01-0.77	0.028	0.12	0.01-0.97	0.048
Patients without triple-negative breast cancer	Histologic differentiation							
	Well/Moderate	79/6						
	Poor	65/13	2.99	1.13-7.87	0.027	2.51	0.93-6.75	0.068
	Pathologic Stage							
	I/II	110/10						
	III/IV	34/9	3.17	1.29-7.82	0.012	1.52	0.58-3.97	0.388
	Surgical procedure							
	Lumpectomy	84/4						
	Mastectomy	61/15	5.65	1.87-17.04	0.002	4.36	1.36-13.95	0.013
	CK1ε							
	<1,5	63/12						
	≥1,5	81/7	0.42	0.16-1.08	0.075	0.39	0.15-1.01	0.051

**Figure 6:**
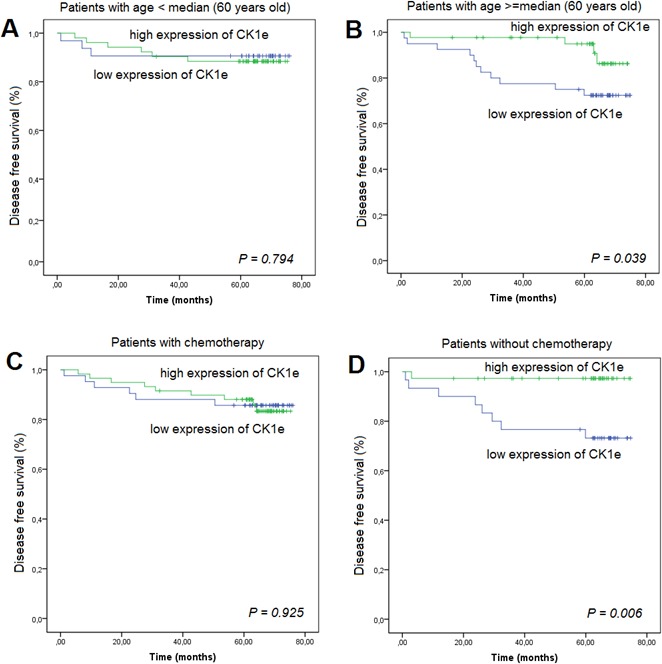
Kaplan-Meier survival curves for breast cancer patients who were classified with either low or high CK1ε expression High CK1ε expression was associated (log-rank) with prognosis as measured by disease-free survival in patients according to age, **A.** below the median, or **B.** equal to or above the median. **C.** adjuvant chemotherapy given or **D.** no adjuvant chemotherapy.

We also analyzed the DFS according to CK1ε expression and age. For patients who had an age > 60 years, those with a higher CK1ε expression had a lower incidence of relapse than those patients with lower expression (*P* = 0.039) (Figure 6B). However, this difference was not significant in younger patients (Figure 6A). Finally, we evaluated the association between CK1ε expression and BC patients treated with or without adjuvant chemotherapy. Strikingly, those patients who did not undergo adjuvant chemotherapy and had a higher CK1ε expression had a higher DFS in the multivariate analyses (*P* = 0.006). However, this difference was not significant in patients who underwent chemotherapy (Figures 6C and 6D).

## DISCUSSION

Our study demonstrates that some subsets of BC patients with high CK1ε expression levels have a lower risk of relapse andhigher DFS. Therefore, increased CK1ε expression is greatly associated with better outcome and might be considered a good survival factor. This is consistent with reports showing that oral squamous cell carcinoma [15] and BC [16] patients who had an increase in CK1ε expression had a considerably better outcome than patients who had lower CK1ε expression. Our data are also in agreement with those of Richter et al [17] for colorectal cancer patients, where high expression of CK1ε was significantly correlated with survival in a cohort of 122 patients. On the contrary, in ovarian cancer, it has been shown that expression of CK1ε is related to poor survival [10]. Fuja et al [16] showed that CK1ε levels were reduced in poorly differentiated tumors and overexpressed in more benign ductal cell carcinoma in situ, thus correlating decreased levels of CK1ε with more aggressive tumors. They hypothesized that the poor prognosis of low levels of CK1ε was more related to the downregulation of CK1ε in tumor tissue, which was also observed in head and neck squamous tumors. We also observed higher levels of CK1ε in noninvasive BC than in infiltrating BC (Figure 3A); however, the levels are similar among invasive BC of different T stages. The fact that CK1ε is a predictive marker independent from tumor differentiation, stage, molecular type, hormone receptors, p53 and Ki67 indicates that the better prognosis of high CK1ε levels is not related to its tumor suppressor properties. We suggest that higher levels of CK1ε activity may sensitize tumors to radiation therapy, perhaps by increasing the proliferation rate, which will correlate with our data (Figure 1) and those reporting CK1ε as an oncogene [5, 6, 14, 17, 18]. The latter is also supported by the development of CK1ε inhibitors that reduced the proliferation index and tumor burden [14, 17, 18] and may sensitize tumor cells to other therapies [4–6]. However, in light of our results of better prognosis in the absence of chemotherapy (Figure 6D), these CK1ε inhibitors should be used very cautiously for treating different tumors and with the different therapeutic modalities.

Additionally, we were able to identify subgroups of BC patients with marginal statistically significant association between higher CK1ε expression and an increase in DFS, which are those having an age ≥ median, Ki67 ≥ median, or wild-type p53 or those without triple-negative breast cancer. Of particular interest were the highly significant differences in DFS according to the CK1ε expression when patients were stratified according to treatment with adjuvant chemotherapy. This observation implies that while BC patients receiving adjuvant chemotherapy have similar outcomes regardless of the CK1ε expression, the absence of this complementary therapy could significantly affect the DFS if the tumor has high levels of CK1ε, and therefore, we can expect a prevalence of relapses for patients with low CK1ε expression. Because this information could be obtained before the initiation of chemotherapy, this additional information could potentially be used as a predictive biomarker to tailor therapy when attempting to devise chemotherapy for an individual patient who has low CK1ε expression. Additional functional studies are required to understand this association.

CK1ε has been shown to be a positive regulator of both the canonical Wnt/β-catenin pathway [5, 19–21] and noncanonical Wnt pathways [22, 23]. WNT, fibroblast growth factor, Notch, Hedgehog, transforming growth factor and the bone morphogenetic protein signaling network are implicated in the maintenance of tissue homeostasis by regulating the self-renewal of normal stem cells as well as the proliferation or differentiation of progenitor cells [24–26]. Disruption of the stem cell signaling network leads to carcinogenesis. In addition, CK1 also suppressed Forkhead O transcription factor (FOXO) activity [27]. Inactivation of FOXO proteins is associated with tumorigenesis, including BC, prostate cancer, glioblastoma, rhabdomyosarcoma and leukemia [28]. Shin et al [29] showed that CK1ε plays a critical role in cancer cell proliferation by controlling mRNA translation. CK1e interacted with and phosphorylated 4E-BP1 at two novel sites, T41 and T50, which were essential for 4E-BP1 inactivation along with increased mRNA translation and cell proliferation. In summary, it has been shown that CK1ε plays a role as a tumor suppressor or oncogene in carcinogenesis. Divergences in pathological diagnosis, tumor size, histologic grade, stage, anatomic location of the tumor, neoadjuvant therapy, etc. among studies may in part explain this apparently contradictory result. CK1ε triggers a diverse array of responses, depending on the genetic makeup and environment of the target cells.

Besides the constraints inherent in any retrospective analysis, our study had several limitations. First, to limit the scope and thus increase the feasibility of this analysis, we selected and analyzed CK1ε expression alone, and we did not find a significant correlation with relapse for the entire group. Occurrence of relapse is likely to involve a complex interplay between several molecular processes that were not analyzed in this study. Second, although we were able to establish strong statistical associations between clinical/pathological factors and the risk of relapse, we did not explore the biological mechanism by which the selected protein led to recurrence in patients with BC. This issue is the topic of ongoing assessment at our institution. In summary, our goal was to identify a biomarker that predicts relapse risk for patients with BC prior to the initiation of therapy. The results from this study demonstrated that low CK1ε expression was associated with poor DFS in patients with BC with negative p53 or poor histologic differentiation or without adjuvant chemotherapy, suggesting the possibility of using this biomarker as a predictive factor for relapse. However, to fully evaluate the significance of the expression of these protein kinases, we will need to both validate the results of this study in a prospective cohort of patients and to understand the effect of this protein on tumor relapse, both of which are currently under investigation.

## MATERIALS AND METHODS

### Patient population

This study was performed in accordance with standard ethical procedures dictated by Spanish law (Ley de Investigación Orgánica Biomédica, 14 July 2007) and was approved by the ethics committee of the Hospital Virgen del Rocío and the Fundación Pública Andaluza para la Gestión de la Investigación en Salud de Sevilla (FISEVI), Spain. Written informed consent was obtained from all patients, and all clinical analyses were conducted in accordance with the principles of the Helsinki Declaration.

Beginning in 2006, 339 patients with a diagnosis of BC were treated with surgery +/− chemo(radio)therapy at our institution. The inclusion criteria were tumor tissue available, no prior thoracic surgery or chemo(radio)therapy, no prior or concurrent other malignancy and no bilateral disease. Ultimately, 168 patients met these criteria. Disease in all cases was staged according to the 2009 (7th) edition of the American Joint Committee on Cancer staging system [30]. The patient data are described in Table 1.

Patients were evaluated at 2 and 6 months and then every year after the completion of treatment. At each visit, a history was taken and a physical examination was performed. Mammography and/or computed axial tomography/magnetic resonance imaging (i.e., after mastectomy) were performed at 6 months after therapy and then yearly. Bone scans or positron emission tomography (PET) scans were performed if clinically indicated.

### Tissue microarray immunohistochemistry

Immunohistochemical studies were performed on breast tumor specimens in a tissue microarray (TMA). The TMA was constructed as previously described [31, 32] and was processed for immunostaining and development according to the vendor's instructions. Two cores per tumor sample were included in the TMA. Incubations with primary antibodies (Anti-CK1epsilon: ab70110 from Abcam; anti-p53: p53 FL 393 (sc-6243) from Santa Cruz) were performed for 40 min. We quantified the levels of CK1ε by immunostaining and by double-blind observation (two independent observers without knowledge of the clinical features of the samples) where the following discrete values were assigned: 0, no CK1ε expression; 1, weak expression; 2, clear strong expression, and 3, very strong expression. Both blind values were averaged for each core sample, and both core sample values were averaged. CK1ε expression determined by the immunohistochemical method was scored as high (≥1.5) or low (<1.5).

### Cell lines and experiments

The T47D cell line was obtained from the ECACC commercial repository at the beginning of this work. No further authentication was conducted by the authors.

For the myristoylated version of the CK1ε protein, the human Src myristoylation sequence was introduced at the 5′ end of the *CSNK1E cDNA* (in a manner similar to that described in [33]).

Cells were cultured by following the experimental procedures in the ATCC cell line data sheet. Retroviral vectors and gene transfers were performed as previously described [32, 34]. Growth curves and colony formation assays were conducted as previously described [32, 34].

### Statistical analysis

Data were analyzed using SPSS (version 19.0) statistical software. Potential risk factors were assessed in univariate analyses using the Cox proportional hazards model. Because of the possible confounding effects of clinical factors on survival, associations found to be statistically significant (*P* < 0.05) or with a marginal trend (*P* < 0.1) in the univariate analysis were adjusted for those variables that had statistical significance on the univariate analysis. The time to end point development was calculated from the surgery date; patients not experiencing the end point were censored at the date of last contact.
